# Supplementary material for: TANK Binding Kinase 1 Promotes BACH1 Degradation through Both Phosphorylation-Dependent and -Independent Mechanisms without Relying on Heme and FBXO22
Source: Int J Mol Sci. 2024 Apr 9;25(8):4141. doi: 10.3390/ijms25084141 (PMC11050367; doi:10.3390/ijms25084141)

Title

TANK binding kinase 1 promotes BACH1 degradation through both phosphorylation-dependent and -independent mechanisms without relying on heme and FBXO22

Liang Liu[1,2], Mitsuyo Matsumoto[1,3], Miki Watanabe-Matsui[4], Tadashi Nakagawa[5,6], Yuko Nagasawa[5], Jingyao Pang[1], Bert K. K. Callens[1,7], Akihiko Muto[1], Kyoko Ochiai[1], Hirotaka Takekawa[1], Mahabub Alam[1,8], Hironari Nishizawa[1], Mikako Shirouzu[9], Hiroki Shima[1,3], Keiko Nakayama[5], Kazuhiko Igarashi[1,3,*]

Supplemental materials and methods

Supplemental references

Supplemental figure legends

Supplemental materials and methods

Cell culture

Human embryonic kidney 293T (HEK-293T) were maintained in DMEM-low glucose supplemented with 10% heat-inactivated FBS (Sigma Aldrich) and 0.1 mg/mL penicillin/streptomycin (Gibco). The cells used were limited to less than 20 passages.

Western blotting

The supernatant containing the proteins was fractionated on slab gels (7.5% or 10% acrylamide separating gel; 4% stacking gel). The gels were run at 100V for 2 hours, and wet-transferred onto polyvinylidene difluoride (PVDF) at 300mA for 1.5 hours at 4 °C. Blots were washed with TBS and blocked for 1 hour in TBS-T/skimmed milk. The primary antibodies were diluted with 5% nonfat dried milk in TBS-T, the membranes were incubated at 4°C for one night. Washes (3 times, 10 min each in TBS-T) were done after primary and secondary antibody incubations. Secondary antibody incubation was performed for 1 hour at RT in 5% nonfat milk in TBS-T. ECL was performed with Clarity Western ECL substrate (Bio-Rad).

Antibodies

Anti-BACH1 mAb (clone 9D11) was purified from the culture supernatant of selected hybridoma. Anti-BACH1 antiserum (A1-6) was reported previously(1). Other antibodies were ACTB (GTX109639, GeneTeX), E-cadherin (ab1416, Abcam), ferritin light chain (sc-74513,Santa Cruz Biotechnology), ferritin heavy chain (sc-376594, Santa Cruz Biotechnology), HO-1 (ADI-SPA-896, Enzo Life Sciences), Anti-DDDDK-

tag(M185-3L, MBL Life Science) and Ubiquitin (MFK-003, Nippon Bio-Test Laboratories Inc).

Mass spectrometry for protein identification

MEL cells were plated at approximately 1 x 10^6 cells per well in 12-well plates and infected with control (FLAG-IL2R) or mBACH1-FLAG-IL2R-expressing retrovirus. ReCLIP was performed using virus-infected MEL cells selected by the antibody of IL2R. Approximately 1×10^8 cells were fixed in PBS containing 0.5 mM DSP and DTME, then lysed in 1 ml of RIPA buffer. Samples were immunoprecipitated using anti-FLAG beads, followed by mass spectrometry. MASCOT was used for protein identification and carried out as described previously (2).

Supplemental references

1. Matsumoto, M., Kondo, K., Shiraki, T., Brydun, A., Funayama, R., Nakayama, K., Yaegashi, N., Katagiri, H., and Igarashi, K. (2016) Genomewide approaches for BACH1 target genes in mouse embryonic fibroblasts showed BACH1-Pparg pathway in adipogenesis. *Genes Cells* **21**, 553-567
2. Li, J., Shima, H., Nishizawa, H., Ikeda, M., Brydun, A., Matsumoto, M., Kato, H., Saiki, Y., Liu, L., Watanabe-Matsui, M., Iemura, K., Tanaka, K., Shiraki, T., and Igarashi, K. (2018) Phosphorylation of BACH1 switches its function from transcription factor to mitotic chromosome regulator and promotes its interaction with HMMR. *Biochem J* **475**, 981-1002

Supplemental figure legends

Supplementary Fig. S1. FBXO22 promotes BACH1 degradation.

A, MEL cells were infected with control (FLAG-IL2R) or mBACH1-Flag-IL2R-expressing retrovirus. ReCLIP was performed using virus-infected MEL cells selected by the antibody of IL2R. Samples were immunoprecipitated using anti-FLAG beads, followed by mass spectrometry. MASCOT was used for protein identification.

B, Namalwa cells were transfected with siRNAs for control (siC) or FBXO22 (siFBXO22) and treated with cycloheximide and hemin for indicated period. Western blots with indicated antibodies are shown.

Supplementary Fig. S2. TBK1 promotes BACH1 degradation.

A and B, HEK293T cells were transfected as written above and then were incubated with CQ (100 μM) or MG132 (10 μM) for 12 hours.

C, HEK293T cells were transfected with the indicated plasmids for 48 hours and were incubated with CQ (100 μM) or MG132 (10 μM) for the last 12 hours. l.ex., long exposure; s.ex., short exposure.

Supplementary Fig. S3. Schematic representations of Plasmids.

A

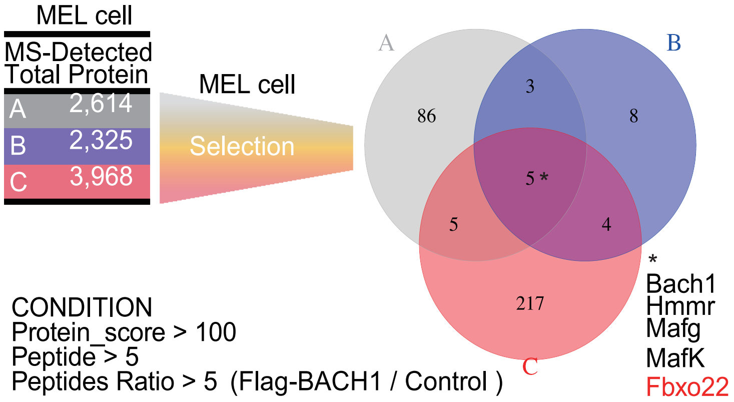
MEL cell
MS-Detected Total Protein
A 2,614
B 2,325
C 3,968
MEL cell
Selection
A
B
C
86
3
8
5 *
5
4
217
*
Bach1
Hmmr
Mafg
MafK
Fbxo22
CONDITION
Protein_score > 100
Peptide > 5
Peptides Ratio > 5 (Flag-BACH1 / Control)

B

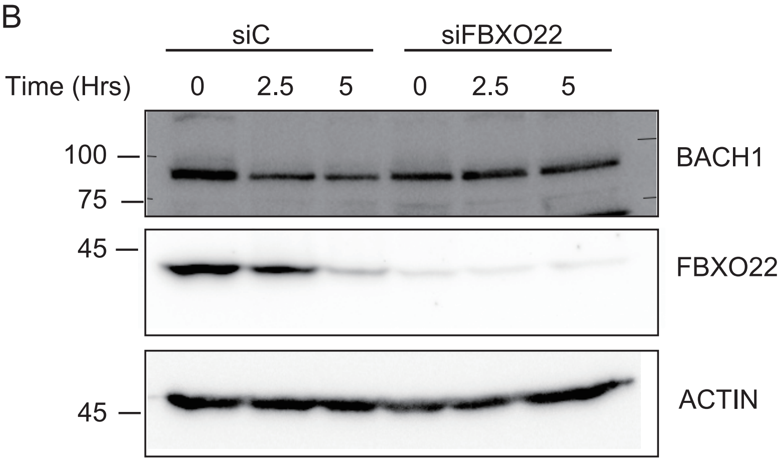
siC
siFBXO22
Time (Hrs)
0
2.5
5
0
2.5
5
100
75
BACH1
45
FBXO22
45
ACTIN

Supplementary Fig. S2

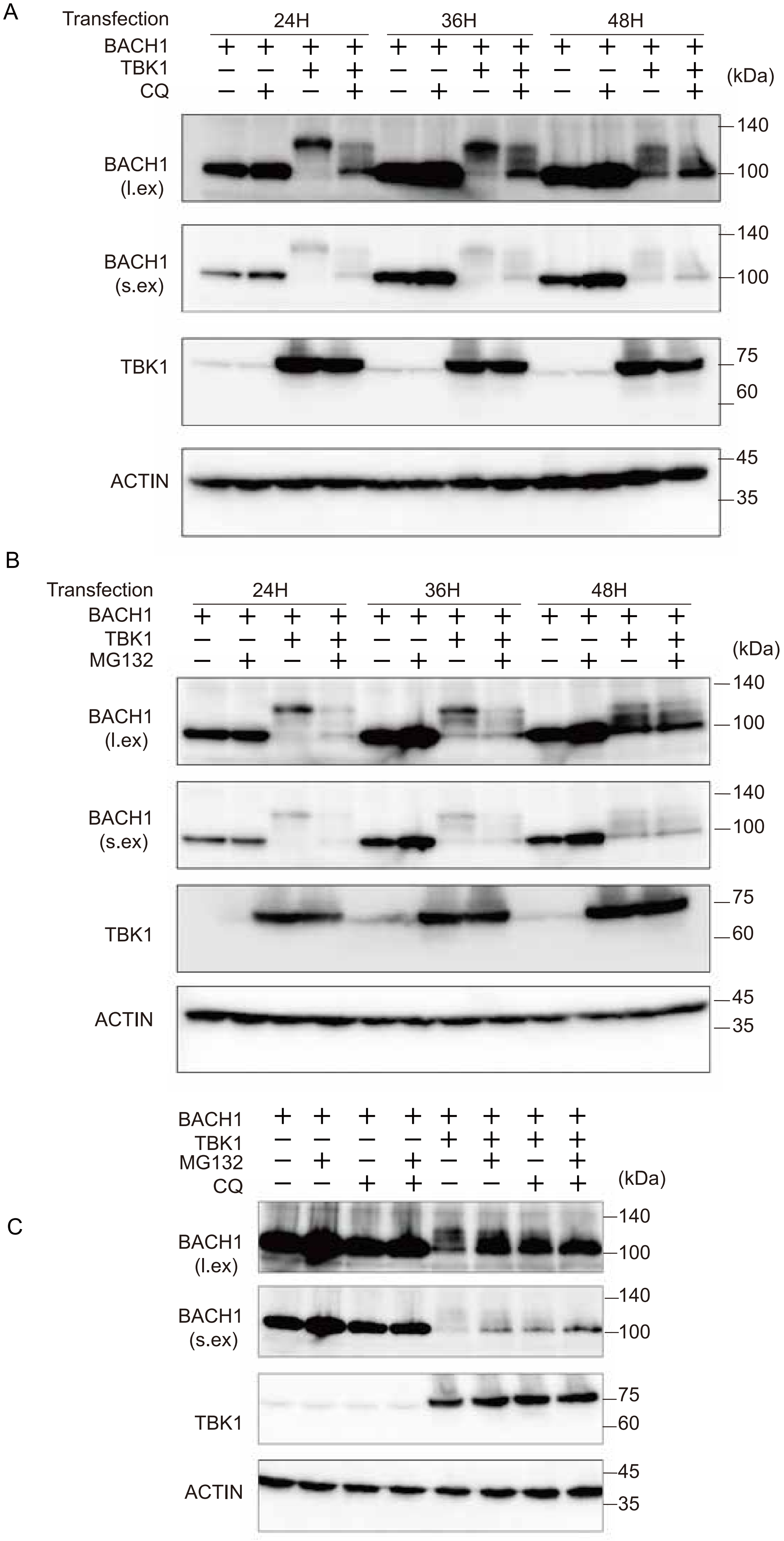

Supplementary Fig. S3

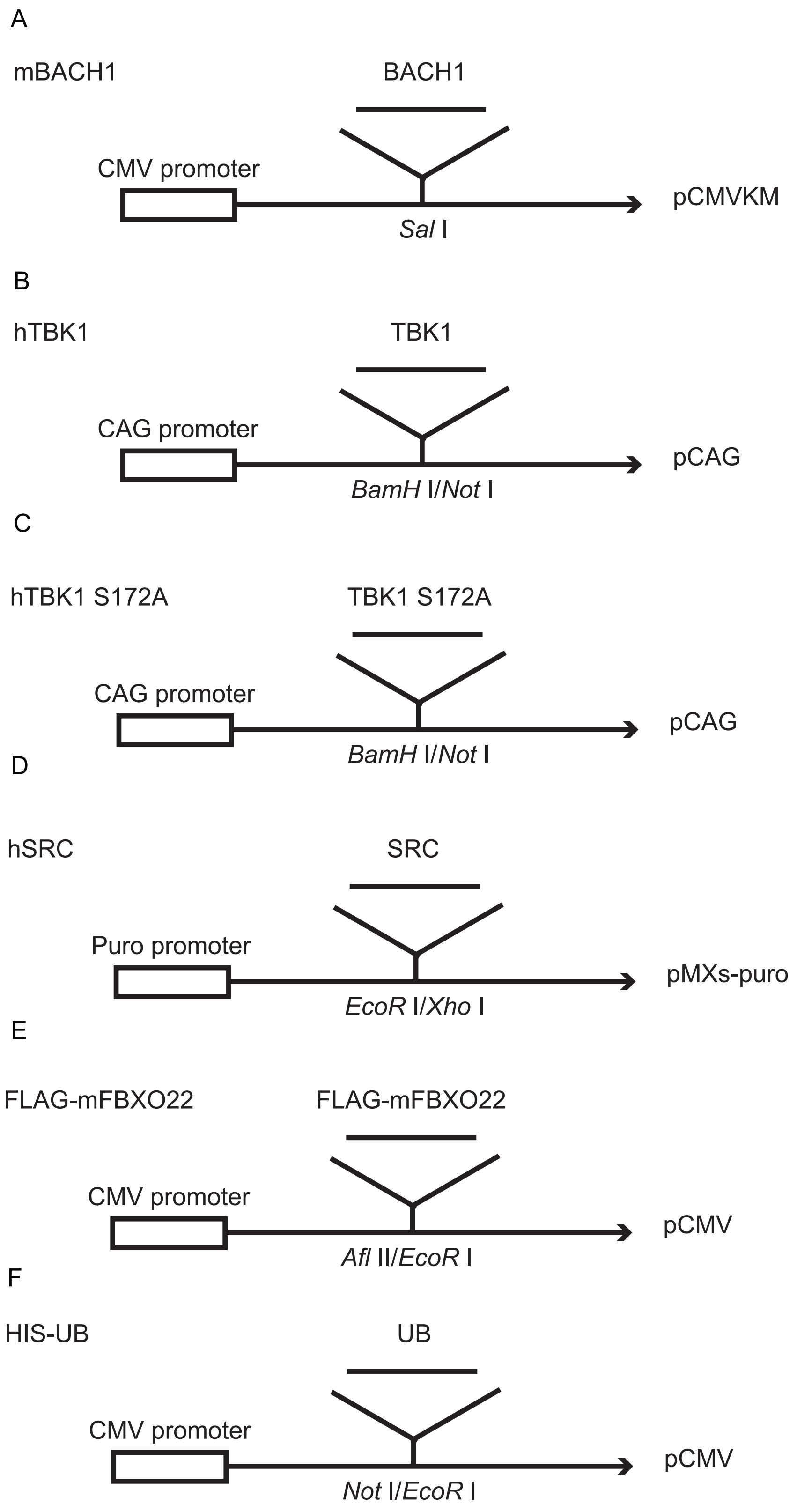

Supplement: Supplementary file 1 [file ijms-25-04141-s001.zip › ijms-2902542-supplementary.pdf]
